# lncRNA LINC00958 Activates NOTCH3 by Competitively Inhibiting miR‐129‐2‐3p to Exacerbate the Malignant Biological Behaviors of Endometrial Cancer Cells

**DOI:** 10.1002/kjm2.70081

**Published:** 2025-10-15

**Authors:** Xia Hong, Su‐Li He

**Affiliations:** ^1^ Department of Obstetrics and Gynecology The Affiliated Hospital of Yangzhou University Yangzhou Jiangsu China

**Keywords:** endometrial cancer, invasion, LINC00958, malignant biological behaviors, migration

## Abstract

Endometrial cancer (EC) is one of the most prevalent gynecologic malignancies, with increasing incidence worldwide. Recent studies have highlighted the critical roles of long noncoding RNAs (lncRNAs) in regulating tumor progression. This study investigated the oncogenic function of lncRNA LINC00958 in EC and its underlying mechanism involving the miR‐129‐2‐3p/NOTCH3 signaling axis. Experimental results showed that LINC00958 was significantly upregulated in EC cell lines. Overexpression of LINC00958 enhanced cell proliferation, migration, and invasion, while inhibiting apoptosis, as evidenced by increased Bcl‐2 and decreased cleaved caspase‐3 and Bax expression. Conversely, silencing LINC00958 suppressed these malignant behaviors. Mechanistically, LINC00958 acted as a competing endogenous RNA (ceRNA), directly binding to miR‐129‐2‐3p and thereby relieving its suppression on NOTCH3. Dual‐luciferase reporter assays confirmed the direct interaction between LINC00958 and miR‐129‐2‐3p, as well as between miR‐129‐2‐3p and NOTCH3. Immunofluorescence analysis further demonstrated enhanced nuclear translocation of NOTCH3 following LINC00958 overexpression. Functional rescue experiments showed that miR‐129‐2‐3p overexpression or NOTCH3 knockdown effectively counteracted the tumor‐promoting effects of LINC00958. In vivo xenograft experiments using Ishikawa cells supported the in vitro findings, confirming that LINC00958 promotes tumor growth by modulating the miR‐129‐2‐3p/NOTCH3 axis. Overall, this study identifies LINC00958 as a novel oncogenic lncRNA in EC, which facilitates tumor progression by sponging miR‐129‐2‐3p and enhancing NOTCH3 signaling. These findings provide new insights into the molecular mechanisms of EC and suggest that targeting the LINC00958/miR‐129‐2‐3p/NOTCH3 axis may represent a promising therapeutic strategy.

## 1 Introduction

Endometrial cancer (EC) represents a leading gynecologic malignancy globally, with a striking increase in incidence over the past two decades. In 2020, the number of newly diagnosed EC cases reached approximately 380,000 worldwide [1, 2, 3]. EC is conventionally categorized into two major types based on pathogenesis and tumor biology: Type I, which is estrogen‐dependent and predominantly composed of endometrioid carcinomas, with a small portion being mucinous adenocarcinomas, and Type II, which is estrogen‐independent and includes clear cell, serous, and other less common histological variants [1, 2, 4]. Although early‐stage EC is often associated with a favorable clinical outcome, the prognosis for advanced, recurrent, or metastatic disease remains poor [5]. Key malignant phenotypes such as cell migration, invasion, and proliferation have been identified as central mechanisms underlying EC progression [6].

Long noncoding RNAs (lncRNAs) are transcripts exceeding 200 nucleotides in length that lack a protein‐coding open reading frame [7]. These RNAs are predominantly transcribed by RNA polymerase II, often producing transcripts longer than 500 nucleotides, and can undergo splicing. lncRNAs are derived from diverse genomic loci, including intronic intergenic regions, and are in antisense relative to protein‐coding genes [8]. One such lncRNA, long intergenic nonprotein coding RNA958 (LINC00958), is significantly upregulated in various human cancers, including head and neck squamous cell carcinoma, hepatocellular cancer, breast cancer, colorectal cancer, bladder cancer, and non–small cell lung cancer, drawing considerable attention in recent literature [9]. Importantly, elevated LINC00958 expression has been documented in EC and is associated with poor patient survival outcomes [10]. microRNAs (miRNAs) are small, noncoding RNAs that modulate gene expression posttranscriptionally. They are central to numerous biological processes, including cell growth, differentiation, apoptosis, and development, and have been implicated in a broad range of diseases. Notably, clinical trials involving miRNA‐based therapies have demonstrated encouraging results, particularly for viral infection and cancer treatment [11]. By targeting specific mRNAs, miRNAs can influence critical signaling cascades involved in tumor progression, including those relevant to EC pathogenesis [12, 13]. Previous studies have shown that miR‐129‐2‐3p is markedly downregulated in EC, and lncRNA XIST has been reported to exert regulatory control over EC progression via the miR‐129‐2‐3p/CCP110 axis [14]. Based on computational predictions using the IntaRNA database, LINC00958 may similarly bind to miR‐129‐2‐3p. Nevertheless, empirical evidence elucidating the functional role of the LINC00958/miR‐129‐2‐3p interactions in the malignant phenotypes of EC remains scarce and warrants further investigation.

The NOTCH signaling pathway, conserved across a broad range of species, is mediated by four canonical receptors (NOTCH1–4) and five membrane‐bound ligands (Jagged1‐2, DLL1, DLL3, DLL4). Upon ligand engagement, NOTCH receptors undergo proteolytic cleavage, leading to the release of the intracellular domain that translocates to the nucleus and modulates the transcription of downstream target genes [15]. Importantly, the NOTCH pathway plays a context‐specific role in tumor biology, functioning either as an oncogene or a tumor suppressor based on factors such as cell type, developmental stage, signal strength, and microenvironmental context [16]. In EC, NOTCH3 has been reported to be markedly overexpressed and implicated in disease progression [17, 18]. Evidence from neurological studies has shown that lncRNA NEAT1 can regulate the NOTCH pathway in epilepsy through miR‐129‐5p, influencing neuroinflammation [19]. Similarly, in esophageal squamous cell carcinoma, hsa_circ_0001741 promotes malignancy via miR‐491‐5p‐mediated upregulation of NOTCH3 [20]. Through bioinformatic prediction using the StarBase database, we identified potential binding interactions between miR‐129‐2‐3p and NOTCH3, indicating a possible posttranscriptional regulatory mechanism. These observations led us to hypothesize that lncRNA LINC00958 may facilitate the malignant phenotype of EC by acting as a competing endogenous RNA for miR‐129‐2‐3p, thereby derepressing NOTCH3 expression. Despite this plausible mechanism, limited empirical evidence exists to clarify the role of the lncRNA LINC00958/miR‐129‐2‐3p/NOTCH3 axis in EC pathogenesis. Therefore, the present study investigates this regulatory cascade, with the objective of identifying LINC00958 and NOTCH3 as potential molecular targets for therapeutic intervention in EC metastasis.

## 2 Materials and Methods

### 2.1 Cell Culture

Human endometrial epithelial cells (hEECs; FH‐H058) and human EC cell lines Ishikawa (FH0305), KLE (FH0304), RL95‐2 (FH0303), Hec‐1A (FH0307), and Hec‐1B (FH0306) were procured from Fuheng Biology (Shanghai, China). Cells were maintained in Dulbecco's modified Eagle medium (DMEM; FH‐D04, Fuheng Biology) supplemented with 10% fetal bovine serum (FBS) and incubated at 37°C in a humidified atmosphere containing 5% CO_2_.

### 2.2 Cell Transfection and Grouping

Various constructs and small RNA oligonucleotides, including pcDNA3.1‐LINC00958 (oe‐LINC00958), LINC00958 siRNA (si‐LINC00958), miR‐129‐2‐3p mimics, mimics negative control (mimics NC), miR‐129‐2‐3p inhibitors, inhibitors NC, NOTCH3 siRNA (si‐NOTCH3), pcDNA3.1‐NC (oe‐NC), and si‐NC, were obtained from GenePharma (Shanghai, China). These were transfected into Ishikawa cells or Hec‐1A cells at a concentration of 100 ng/μL using Lipofectamine 2000 (Invitrogen, Carlsbad, CA, USA) following the manufacturer's instructions. Experiments were performed 48 h post‐transfection. The oligonucleotide sequences employed were as follows: mimics NC: 5′‐UUGUCCGAACGUGUCACGUTT‐3′; miR‐129‐2‐3p mimics: 5′‐AAGCCCUUACCCCAAAAAGCAU‐3′; inhibitors NC: 5′‐CACUACUUUUGUGUAGUACAA‐3′; miR‐129‐2‐3p inhibitors: 5′‐AUGCUUUUUGGGGUAAGGGCUU‐3′; si‐LINC0095: 5′‐GAGAAAGTTTAAGCTCTCCTCACTA‐3′; LINC0095 NC: 5′‐GAGTGAATTCGATCTTCCACAACTA‐3′; si‐NOTCH3: 5′‐CAGGCGAGAGCTGCAGTCAGAATAT‐3′; NOTCH3 NC: 5′‐CAGGAGCGACGTTGAGACAAGCTAT‐3′.

Cells were divided into the following groups: (1) the Blank group (Ishikawa/Hec‐1A cells cultured under standard conditions); (2) the si‐NC group (Ishikawa cells transfected with si‐NC for 48 h); (3) the si‐LINC00958 group (Ishikawa cells transfected with si‐LINC00958 48‐h); (4) the oe‐NC group (Hec‐1A cells transfected with oe‐NC for 48 h); (5) the oe‐LINC00958 group (Hec‐1A cells transfected with oe‐LINC00958 for 48 h); (6) the oe‐LINC00958 + mimics group (Hec‐1A cells co‐transfected with oe‐LINC00958 and mimics NC for 48 h); (7) the oe‐LINC00958 + miR‐129‐2‐3p mimics group (Hec‐1A cells co‐transfected with oe‐LINC00958 and miR‐129‐2‐3p mimics for 48 h); (8) the oe‐LINC00958 + si‐NC group (Hec‐1A cells co‐transfected with oe‐LINC00958 and si‐NC for 48 h); (9) the oe‐LINC00958 + si‐NOTCH3 group (Hec‐1A cells co‐transfected with oe‐LINC00958 and si‐NOTCH3 for 48 h). All experiments were independently repeated three times.

### 2.3 Reverse Transcription Quantitative Polymerase Chain Reaction (RT‐qPCR)

Total RNA was extracted using TRIzol reagent (Invitrogen) and reverse‐transcribed into complementary DNA using PrimeScript RT reagent kit (TaKaRa, Kyoto, Japan). TaqMan primers and probes were also sourced from TaKaRa. Quantitative polymerase chain reaction (qPCR) was performed on an ABI PRISM 7900 sequence using SYBR Green II (TaKaRa). The thermal cycling conditions included an initial denaturation at 95°C for 5 min, followed by 40 cycles of 15‐s denaturation at 95°C, 20‐s annealing at 60°C, and 35‐s extension at 72°C. β‐Actin was used as an internal reference. Relative gene expression levels were analyzed using the $2^{-∆∆C_{t}}$ method. The primer sequences (Sangon, Shanghai, China) are provided in Table 1.

**TABLE 1:** Primer sequences.

Gene	Forward 5′–3′	Reverse 5′–3′
LINC00958	AAAGCAAGGTCTCCCCACAAG	GGTCTGTGCTAGATCAAAAGGCA
NOTCH3	GCCACAGACTGGATGGACAC	CGGATGTCAGCAGCAACCA
miR‐129‐2‐3p	TTCCAAGCCCTTACCCCA	CACTTCCTCAGCACTTGTTCCTAT
U6	ATTGGAACGATACAGAGAAGATT	GGAACGCTTCACGAATTTG
β‐Actin	CATGTACGTTGCTATCCAGGC	CTCCTTAATGTCACGCACGAT

### 2.4 Western Blot

Total cellular protein was extracted by lysing cells in cell lysis buffer, and the concentration of the extracted protein was quantified using the bicinchoninic acid kit (AR1189; Boster, Wuhan, Hubei, China), in accordance with the manufacturer's instructions. Following the addition of an appropriate amount of loading buffer, protein samples were denatured by boiling for 5 min. Denatured protein samples were then subjected to sodium dodecyl sulfate–polyacrylamide gel electrophoresis for separation, and subsequently transferred to polyvinylidene fluoride membranes. The membranes were blocked with 3% bovine serum albumin (BSA) for 2 h at room temperature to prevent nonspecific binding. Primary antibody incubation was performed overnight at 4°C using antibodies (Abcam, Cambridge, UK) against NOTCH3 (1:1000, ab23426), cleaved caspase‐3 (1:2000, ab2302), Bcl‐2 (1:2000, ab182858), and Bax (1:1000, ab32503) at 4°C. Thereafter, membranes were incubated for 1 h at room temperature in the dark with goat anti‐rabbit immunoglobulin G (IgG) H&L (horseradish peroxidase [HRP]) (1:2000, ab6721; Abcam). Protein bands were detected using enhanced chemiluminescence (AR1191, Boster), and grayscale quantification of band intensities was conducted using Image Pro Plus 6.0 (Media Cybernetics, Silver Spring, MD, USA). β‐Actin (1:1000, ab8226; Abcam) served as an internal reference. All Western blot experiments were performed in triplicate.

### 2.5 Cell Counting Kit‐8 Assay

Cell proliferation was assessed using the Cell Counting Kit‐8 (CCK‐8) kit (AR1199; Boster). After transfection, cells were seeded into 96‐well plates at a density of 1 × 10^4^ cells per well and incubated for 48 h. Subsequently, 10 μL of CCK‐8 reagent was added to each well, followed by 2 h of incubation at 37°C in a humidified incubator with 5% CO_2_. The optical density (OD) at 450 nm was measured using a microplate reader (Tecan, Mannedorf, Switzerland). The cell survival rate was calculated using the formula: [(OD_450_ of sample − OD_450_ of the blank control)/(OD_450_ of the negative control − OD_450_ of the blank control)] × 100%.

### 2.6 Transwell and Wound Healing Assays

A Transwell assay was performed to assess cell invasion capacity. Briefly, 5 × 10^4^ cells suspended in 200 μL of serum‐free medium were seeded into the upper chamber of Transwell plates (BD Biosciences, San Diego, CA, USA) pre‐coated with Matrigel. The lower chamber was filled with 600 μL of medium containing 10% FBS as a chemoattractant. Following 24‐h incubation, the invasive cells on the lower surface were fixed with methanol for 20 min and stained with a crystal violet solution (0.1%). Cells were visualized and counted under a light microscope (TS100, Nikon, Tokyo, Japan) [21].

Cell migration was assessed using a wound healing assay. Cells were seeded into six‐well plates (1 × 10^4^ cells per well) and allowed to reach 80%–90% confluence. A linear scratch was made using a P‐200 pipette tip, followed by incubation in serum‐free DMEM. Images were captured at 0 and 24 h using an inverted microscope (TS100, Nikon), and wound closure was quantified by calculating the percentage reduction in wound width over time [21].

### 2.7 Apoptosis Assessment by Flow Cytometry

The cells were rinsed three times with pre‐cooled phosphate buffer saline (PBS), resuspended in 0.3 mL PBS, and fixed in 0.7 mL absolute ethyl alcohol. Fixed cells were stored at −20°C for 24 h. Following centrifugation at 1000 r/min for 15 min, the ethanol was discarded, and the cells were washed twice with PBS. Subsequently, cells were incubated with 120 μL (200 μg/mL) RNaseA for 30 min at 37°C and stained with propidium iodide (PI) staining solution (Beyotime) and annexin V‐fluorescein isothiocyanate (Biosciences, San Jose, CA, USA).

A flow cytometry assay was performed using the FACS Aria II Cell Sorter (BD Biosciences), and data analysis was conducted using FlowJo software (TreeStar, Ashland, OR, USA).

### 2.8 Immunofluorescence Assay

The prepared cell slides were washed three times with PBS and fixed in 4% polymerase at room temperature for 30 min. Following fixation, the samples were gently agitated in PBS (three times for 5 min each) at room temperature. Cells were permeabilized with 0.1% Triton X‐100 for 30 min, then blocked with BSA (1%) for 30 min at room temperature. After additional PBS washes, the slides were incubated overnight at 4°C with primary antibodies against NOTCH3 (1:200, ab23426; Abcam). Subsequently, cells were incubated in the dark with a fluorescent secondary antibody (CoraLite488; 1:50) at room temperature for 1.5 h. Nuclear staining was performed using 4′,6‐diamidino‐2‐phenylindole for 30 min. After three final PBS washes, the samples were imaged under a fluorescence microscope (Olympus, Tokyo, Japan).

### 2.9 Dual‐Luciferase Reporter Gene Assay

Potential binding interactions between LINC00958 and miR‐129‐2‐3p, and between miR‐129‐2‐3p and NOTCH3 were predicted using the IntaRNA 2.0 database (http://rna.informatik.uni‐freiburg.de/IntaRNA/Input.jsp) or the StarBase database (https://rnasysu.com/encori/). Wild‐type and mutant sequences of LINC00958 or NOTCH3 containing the putative miR‐129‐2‐3p binding sites were cloned into the PMIRGLO Reporter Vector (Promega Corporation, Madison, WI, USA). These reporter constructs co‐transfected with miR‐129‐2‐3p mimics, or mimics NC, were co‐transfected with LINC00958‐WT/MUT and NOTCH3‐WT/MUT reporter vectors into Ishikawa or Hec‐1A cells using the Lipofectamine 2000 assay kit. After 48 h, luciferase activity was measured using the dual‐luciferase gene assay system (Promega).

### 2.10 Establishment of EC Mouse Model

A total of 18 BALB/c nude mice (weight: 20 ± 2 g) were obtained from Genhouse Bio Co. Ltd., Suzhou, China, and housed in a specific pathogen‐free facility under standard conditions (24°C ± 2°C, 50% ± 10% humidity, 12‐h light–dark cycle).

Lentiviral vectors encoding oe‐LINC00958 lentivirus (Lv‐oe‐LINC00958) and its NC (Lv‐oe‐NC) (GenePharma), both at a final concentration of 100 ng/μL, were transfected into Ishikawa cells using Lipofectamine 2000 (Invitrogen).

BALB/c nude mice were randomly assigned into five groups (six mice/group): (1) Model group (1 × 10^7^ Ishikawa cells were subcutaneously inoculated on the right side of BALB/c nude mice) [22]; (2) Lv‐oe‐NC group (1 × 10^7^ Ishikawa cells transfected with Lv‐oe‐NC were subcutaneously inoculated on the right side of BALB/c nude mice); (3) Lv‐oe‐LINC00958 group (1 × 10^7^ Ishikawa cells transfected with Lv‐oe‐LINC00958 were inoculated subcutaneously on the right side of BALB/c nude mice); (4) Lv‐oe‐LINC00958 + agomir NC group (1 × 10^7^ Ishikawa cells co‐transfected with Lv‐oe‐LINC00958 and agomir NC were subcutaneously inoculated on the right side of BALB/c nude mice); (5) Lv‐oe‐LINC00958 + miR‐129‐2‐3p agomir group (1 × 10^7^ Ishikawa cells co‐transfected with Lv‐oe‐LINC00958 and miR‐129‐2‐3p agomir were subcutaneously inoculated on the right side of BALB/c nude mice).

Two weeks postinoculation, the mice were euthanized via intraperitoneal injection of 0.5% pentobarbital sodium (150 mg/kg; P3761, Sigma‐Aldrich, St. Louis, MO, USA), and tumor tissues were harvested. Tumor volume was calculated using the formula: π/6 × length × width^2^ [23]. Half of the excised tumor tissues from each mouse were fixed in paraffin for histological evaluation, while the remaining half was homogenized and stored at −80°C for subsequent analysis.

### 2.11 Immunohistochemistry Assay

Paraffin‐embedded tumor tissue sections were rinsed with PBS and subjected to antigen retrieval by boiling in 0.01 M sodium citrate buffer (Sigma‐Aldrich). Endogenous peroxidase activity was blocked with 3% H_2_O_2_ for 15 min at room temperature. Following a 30‐min blockade with 5% goat serum (SL038; Solarbio, Beijing, China), sections were incubated overnight at 4°C with primary antibodies against NOTCH3 (1:100, ab52627; Abcam) or anti‐Ki67 (5 μg/mL, ab15580; Abcam). Subsequently, sections were incubated with the secondary antibody goat anti‐rabbit IgG H&L (HRP) (1:1000, ab214050; Abcam) for 60 min at 37°C, visualized using diaminobenzidine (DA1016; Solarbio), and counter‐stained with hematoxylin (H8070; Solarbio). Imaging was performed using an Olympus microscope (Olympus), and quantitative analysis was conducted with Image Pro Plus 6.0 (Media Cybernetics) [24].

### 2.12 Statistical Analysis

Statistical analysis and graphing were performed using GraphPad Prism 9.5 (GraphPad, San Diego, CA, USA). The normality of continuous variables was assessed using the Kolmogorov–Smirnov test. Normally distributed data were expressed as mean ± standard deviation. One‐way analysis of variance (ANOVA) followed by Tukey's multiple comparison test was employed for comparisons among multiple groups. A *p* < 0.05 was considered statistically significant.

## 3 Results

### 3.1 LINC00958 Promoted Malignant Biological Behaviors of EC Cells

**FIGURE 1:**
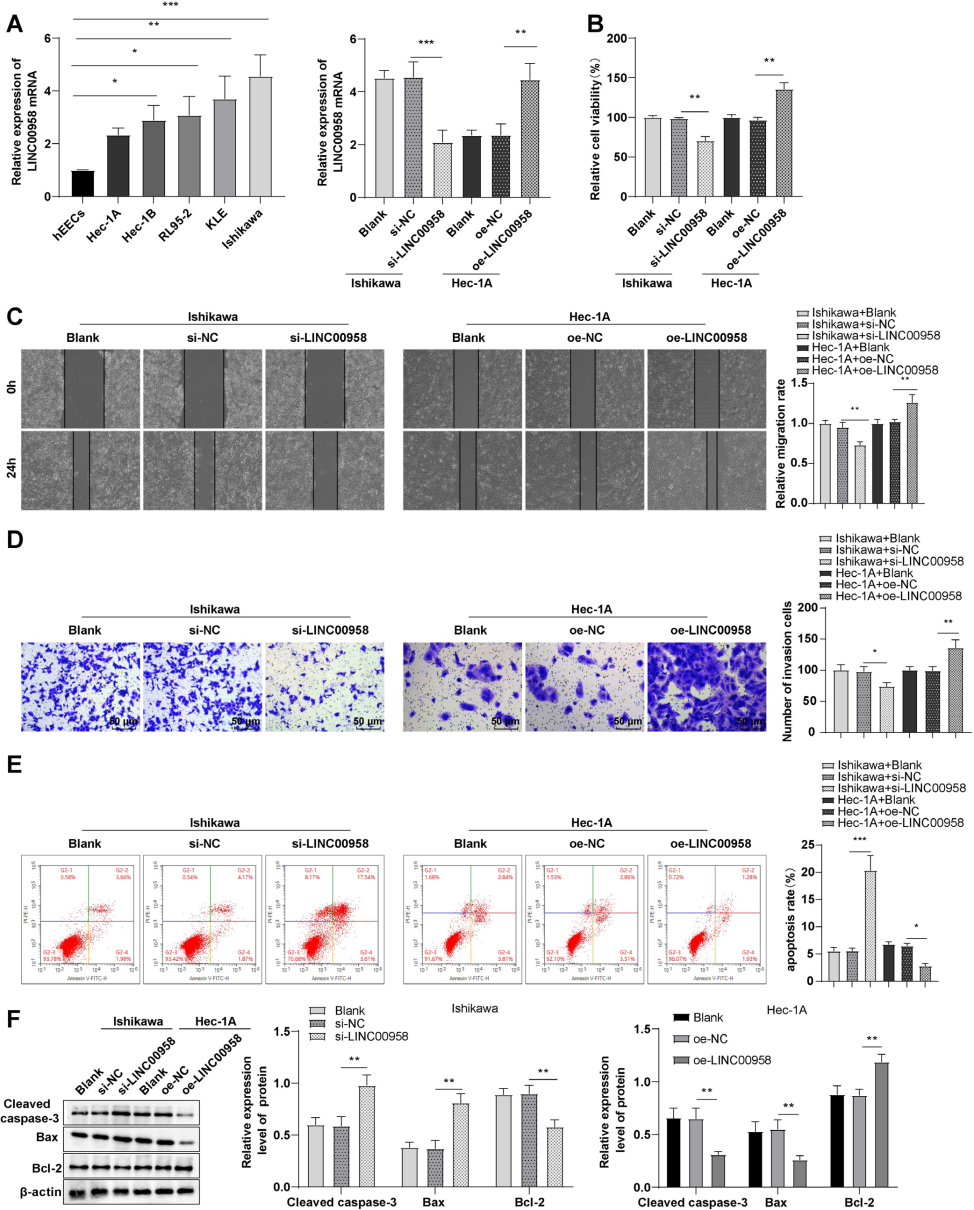
LINC00958 promoted the malignant biological behaviors of EC cells. (A) RT‐qPCR to measure the LINC00958 mRNA level. (B) CCK‐8 assay to assess cell viability. (C) Wound healing assay to evaluate cell migratory rate. (D) Transwell assay to evaluate cell invasion. (E) Flow cytometry assay to assess cell apoptosis. (F) Western blot to determine apoptosis‐related protein levels (cleaved caspase‐3, Bcl‐2, Bax). The experiments were repeated three times. Data were expressed as mean ± standard deviation. One‐way ANOVA was applied for data comparisons among multiple groups, followed by Tukey's test. *p < 0.05, **p < 0.01, ***p < 0.001.

RT‐qPCR analysis demonstrated that the expression of LINC00958 was significantly upregulated in five EC cell lines (RL95‐2, KLE, Hec‐1A, Hec‐1B, and Ishikawa) relative to hEECs (Figure 1A, all *p* < 0.05). Among these, the Ishikawa cell line exhibited the highest relative expression level of LINC00958, whereas the Hec‐1A cell line showed comparatively lower expression. Therefore, Ishikawa and Hec‐1A cells were selected for use in subsequent mechanistic and functional analyses.

LINC00958 was successfully knocked down in Ishikawa cells using si‐LINC00958 transfection and overexpressed in Hec‐1A cells via oe‐LINC00958 transfection (Figure 1A, all *p* < 0.01). Silencing of LINC00958 reduced cell viability, migration, and invasion, increased apoptosis (evidenced by elevated cleaved caspase‐3, Bax levels, diminished Bcl‐2 level, and higher apoptotic cell count), whereas LINC00958 overexpression produced the opposite effects (Figure 1B–F, all *p* < 0.05). The findings indicate that LINC00958 is upregulated in EC cells and promotes malignant biological behaviors, which can be suppressed through its knockdown.

### 3.2 LINC00958 Targeted miR‐129‐2‐3p

**FIGURE 2:**
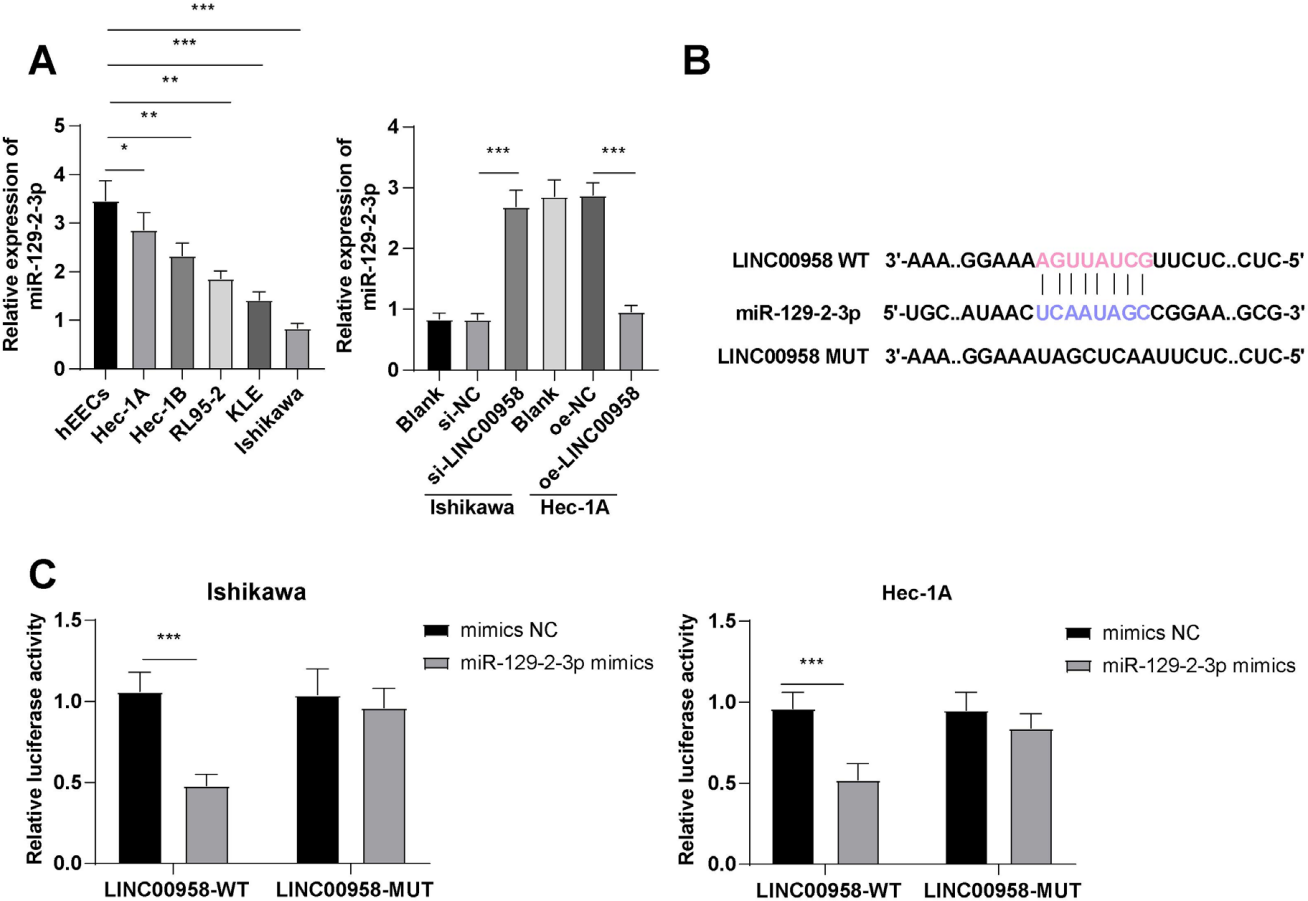
LINC00958 targeted miR‐129‐2‐3p. (A) RT‐qPCR to determine miR‐129‐2‐3p expression. (B) The IntaRNA 2.0 database predicted the binding sites of LINC00958 to miR‐129‐2‐3p. (C) The dual‐luciferase assay confirmed the targeted binding relationship between LINC00958 and miR‐129‐2‐3p. The experiments were repeated three times. Data were expressed as mean ± standard deviation. The independent sample *t*‐test was used to compare the data between two groups. One‐way ANOVA was used for comparing data between multiple groups, and Tukey's test for post hoc testing. *p < 0.05, **p < 0.01, ***p < 0.001.

To determine whether LINC00958 targets miR‐129‐2‐3p in promoting EC cell malignancy, RT‐qPCR analysis revealed significantly reduced miR‐129‐2‐3p expression in EC cell lines compared to controls (Figure 2A, all *p* < 0.05). Notably, LINC00958 knockdown increased miR‐129‐2‐3p levels, whereas its overexpression led to a significant reduction (Figure 2A, all *p* < 0.001). Bioinformatic analysis using the IntaRNA 2.0 database predicted potential binding sites between LINC00958 and miR‐129‐2‐3p (Figure 2B). Dual‐luciferase assays further confirmed a direct binding relationship (Figure 2C, all *p* < 0.001), supporting the conclusion that LINC00958 directly targets miR‐129‐2‐3p.

### 3.3 Reexpression of miR‐129‐2‐3p Partially Abrogated the Regulatory Effect of LINC00958 on EC Cells

**FIGURE 3:**
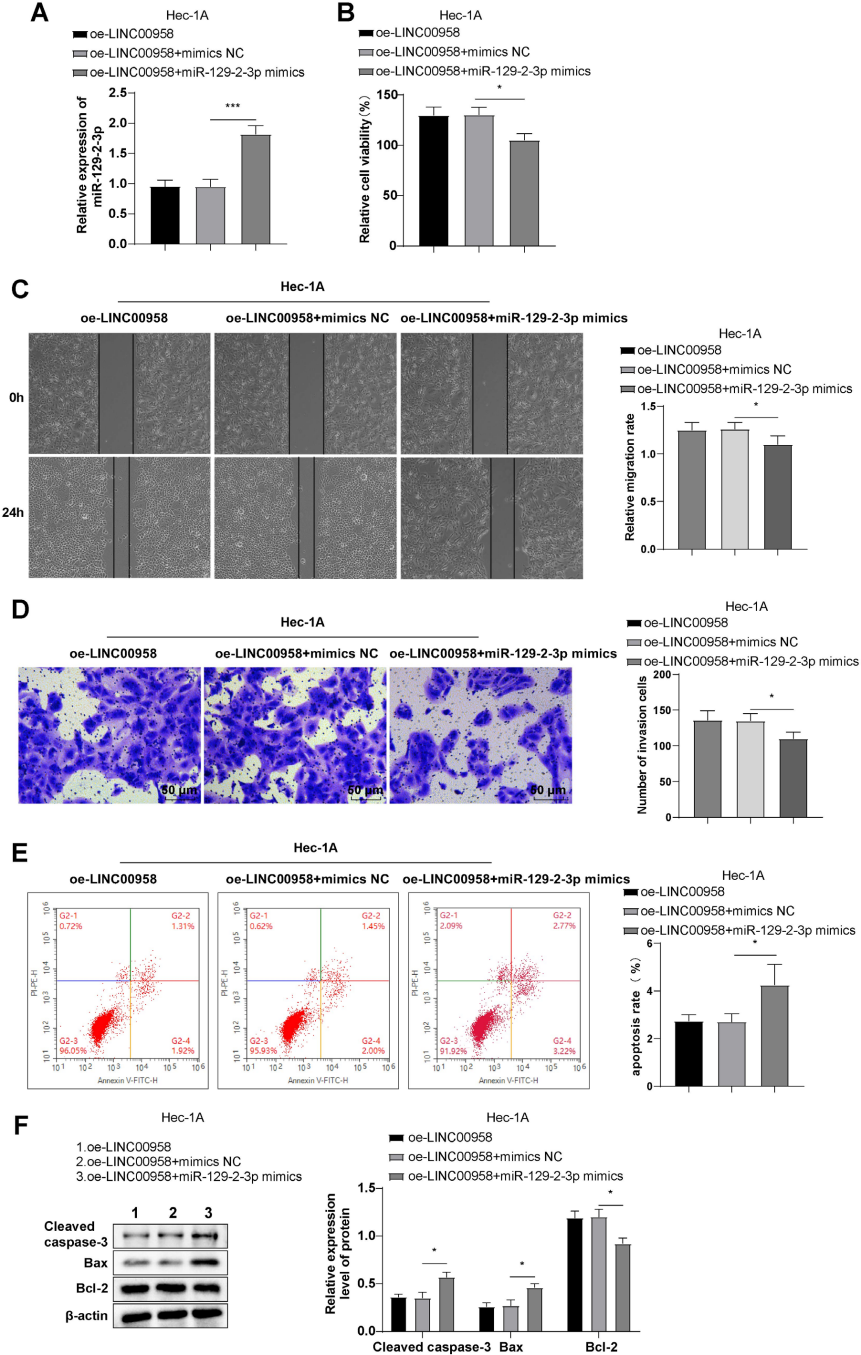
Overexpression of miR‐129‐2‐3p partially annulled the regulatory effect of LINC00958 on EC cells. (A) RT‐qPCR to determine miR‐129‐2‐3p expression. (B) CCK‐8 assay to evaluate cell viability. (C) Wound healing assay to evaluate cell migratory rate. (D) Transwell assay to assess cell invasion. (E) Flow cytometry to assess cell apoptosis. (F) Western blot to measure the expression levels of apoptosis‐related proteins cleaved caspase‐3, Bcl‐2, and Bax. The experiments were repeated three times. Data were expressed as mean ± standard deviation. One‐way ANOVA was applied for data comparisons among multiple groups, followed by Tukey's test. *p < 0.05, ***p < 0.001.

Subsequently, Hec‐1A cells were co‐transfected with oe‐LINC00958 and miR‐129‐2‐3p mimics. Compared to the oe‐LINC00958 + mimics NC group, cells co‐transfected with miR‐129‐2‐3p mimics exhibited elevated miR‐129‐2‐3p expression, reduced cell viability, migration, and invasion, increased apoptosis, elevated Bax and cleaved caspase‐3 levels, and decreased Bcl‐2 expression (Figure 3A–F, all *p* < 0.05). These findings suggest that reexpression of miR‐129‐2‐3p partially counteracts the oncogenic effects of LINC00958 on EC cells.

### 3.4 LINC00958 Facilitated NOTCH3 Expression by Sponging miR‐129‐2‐3p, Thus Stimulating NOTCH3 Nuclear Translocation

**FIGURE 4:**
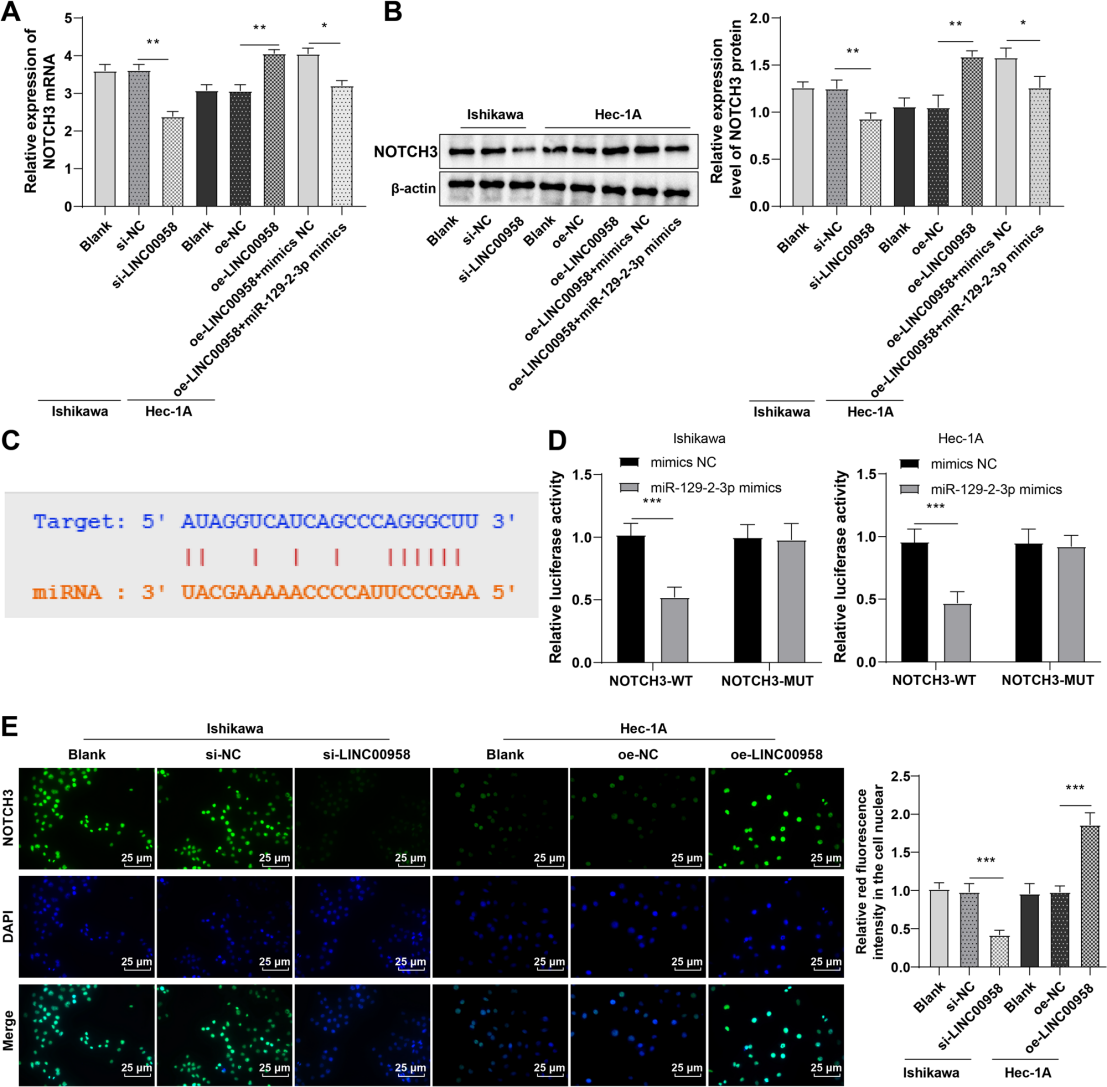
LINC00958 upregulated NOTCH3 by sponging miR‐129‐2‐3p, thus promoting NOTCH3 nuclear translocation. (A) RT‐qPCR to measure NOTCH3 mRNA expression. (B) Western blot to determine NOTCH3 protein expression. (C) StarBase database to predict the targeted binding between miR‐129‐2‐3p and NOTCH3. (D) Dual‐luciferase assay to verify the binding of miR‐129‐2‐3p to NOTCH3. (E) Immunofluorescence to detect nuclear translocation of NOTCH3. The experiments were repeated three times. Data were presented as mean ± standard deviation. One‐way ANOVA was conducted for data comparisons among multiple groups, and Tukey's test for post hoc analysis. *p < 0.05, **p < 0.01, ***p < 0.001.

RT‐qPCR and Western blot analyses revealed that NOTCH3 expression was downregulated in the si‐LINC00958 group compared to the si‐NC group, while it was significantly upregulated in the oe‐LINC00958 group, relative to the oe‐NC group. Co‐transfection of LINC00958 with miR‐129‐2‐3p mimics attenuated this upregulation (Figure 4A,B, all *p* < 0.05). StarBase database predictions identified putative binding sites between miR‐129‐2‐3p and NOTCH3 (Figure 4C), which were subsequently validated through dual‐luciferase assay (Figure 4D, all *p* < 0.001). Immunofluorescence assay demonstrated reduced nuclear localization of NOTCH3 in the si‐LINC00958 group and increased nuclear NOTCH3 in the oe‐LINC00958 group, relative to their respective controls (Figure 4E, all *p* < 0.001). These findings suggest that LINC00958 upregulates NOTCH3 by sponging miR‐129‐2‐3p, thereby promoting NOTCH3 nuclear translocation in EC cells.

### 3.5 Knockdown of NOTCH3 Partially Reversed the Promotional Actions of LINC00958 on EC Cell Malignant Biological Behaviors

**FIGURE 5:**
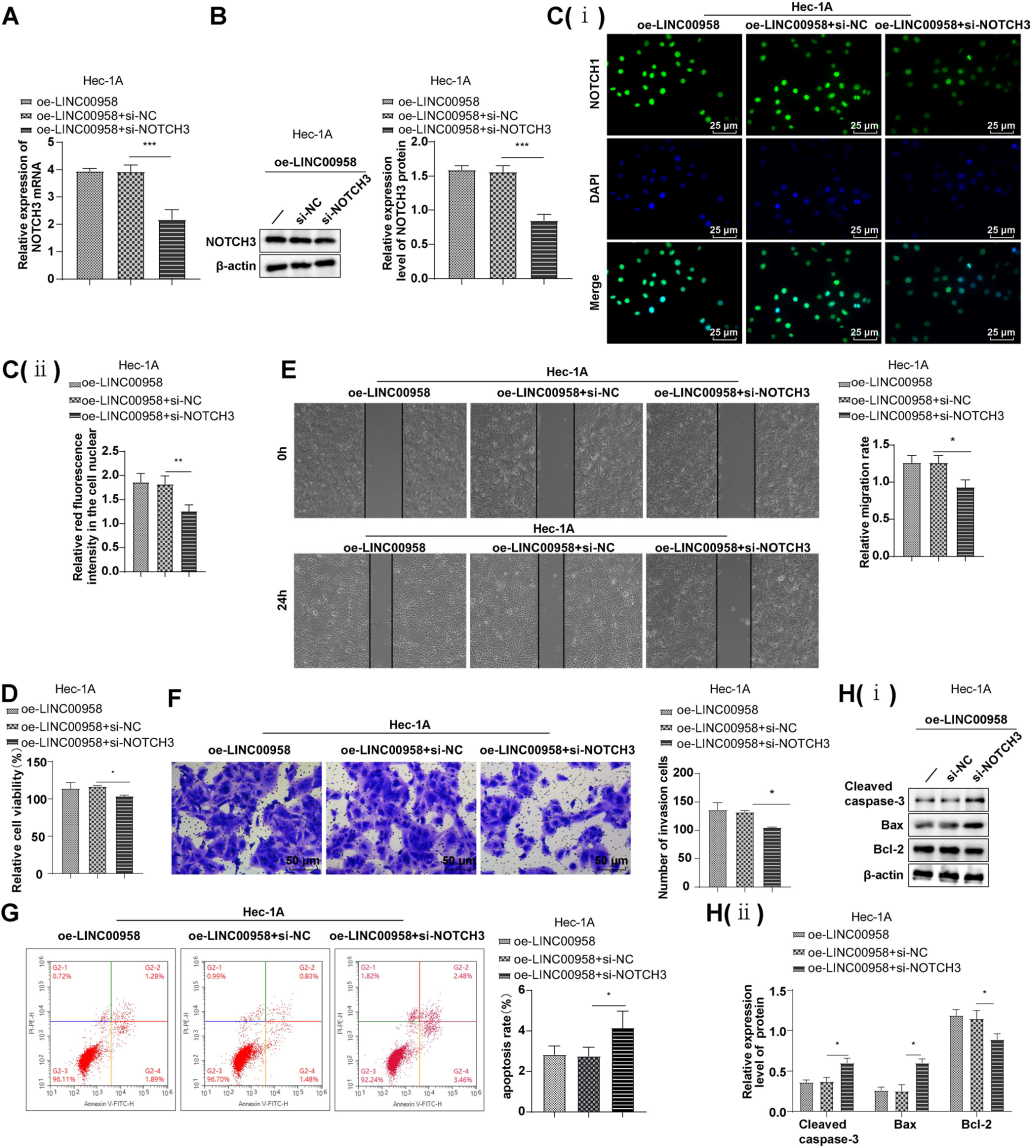
Knockdown of NOTCH3 partially nullified the stimulatory effects of LINC00958 on malignant biological behaviors of EC cells. (A) RT‐qPCR to determine the NOTCH3 mRNA level. (B) Western blot to test NOTCH3, cleaved caspase‐3, Bcl‐2, and Bax protein levels. (Ci) Immunofluorescence to detect the nuclear translocation of NOTCH3 and (Cii) corresponding quantitative analysis. (D) CCK‐8 to assess cell viability. (E) Wound healing assay to evaluate cell migratory rate. (F) Transwell assay to assess cell invasion. (G) Flow cytometry to assess apoptosis. (Hi) Western blot analysis of cleaved caspase‐3, Bcl‐2, and Bax protein expression levels and (Hii) corresponding quantitative analysis. The experiments were conducted in triplicate, and data were presented as the mean ± standard deviation. To compare the data among multiple groups, one‐way ANOVA was used, followed by Tukey's test for post hoc analysis. *p < 0.05, ***p < 0.001.

Hec‐1A cells were co‐transfected with oe‐LINC00958 and si‐NOTCH3. Compared to the oe‐LINC00958 + si‐NOTCH3 group, cells in the oe‐LINC00958 + si‐NOTCH3 group exhibited significantly reduced NOTCH3 expression and nuclear localization, along with decreased cell viability, migration, and invasion. In addition, levels of proapoptotic proteins Bax and cleaved caspase‐3 were elevated, Bcl‐2 expression was suppressed, and the apoptotic cell number was increased (Figure 5A–H, all *p* < 0.05). These findings indicate that NOTCH3 knockdown partially abrogated the pro‐tumorigenic effects of LINC00958 on EC cells.

### 3.6 LINC00958 Modulated the miR‐129‐2‐3p/NOTCH3 Axis to Promote EC Growth In Vivo

**FIGURE 6:**
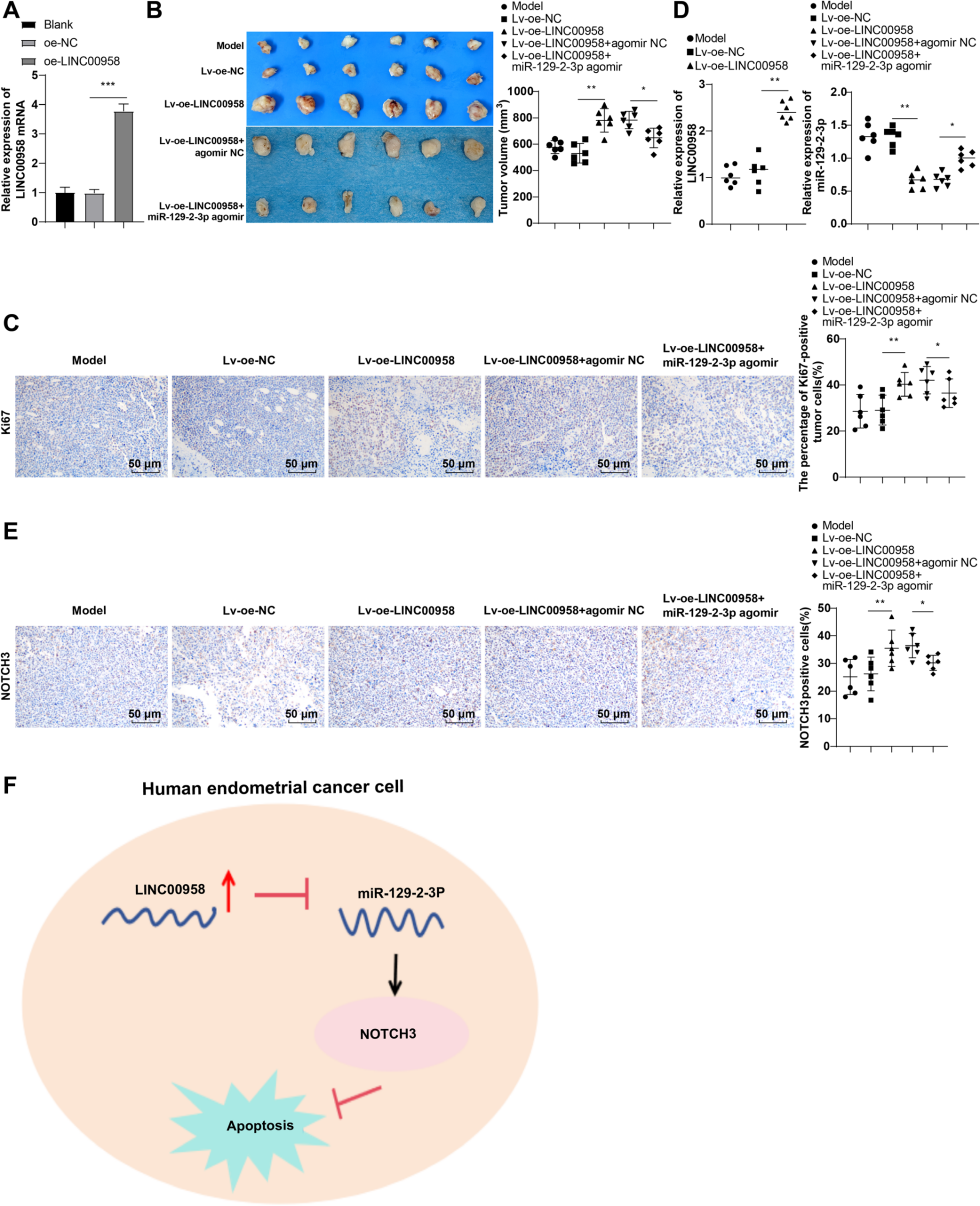
LINC00958 regulated the miR‐129‐2‐3p/NOTCH3 axis to promote EC progression in vivo. (A) RT‐qPCR to determine LINC00958 expression in cells (*n* = 3). (B) Representative images of tumor size and volume graphs (*n* = 6). (C) Ki67 IHC assay to assess tumor proliferation (*n* = 6). (D) RT‐qPCR to measure LINC00958 or miR‐129‐2‐3p expression (*n* = 6). (E) IHC to determine the protein level of NOTCH3 in tissues (*n* = 6). (F) Regulatory effects of LINC00958 on miR‐129‐2‐3p and NOTCH3. Data were expressed as mean ± standard deviation, and one‐way ANOVA was conducted for data comparisons among multiple groups, followed by Tukey's multiple comparison test. *p < 0.05, **p < 0.01, ***p < 0.001.

Following Lv‐oe‐LINC00958 transfection, LINC00958 expression in Ishikawa cells was significantly upregulated (Figure 6A, *p* < 0.001), accompanied by a marked increase in tumor volume in vivo (Figure 6B, *p* < 0.001). Immunohistochemistry (IHC) analysis revealed enhanced Ki67‐positive cell staining in the Lv‐oe‐LINC00958 group compared to the Lv‐oe‐NC group, indicating increased tumor cell proliferation (Figure 6C, *p* < 0.05). RT‐qPCR showed elevated LINC00958 expression and reduced miR‐129‐2‐3p levels in tumor tissues from the Lv‐oe‐LINC00958 group (*p* < 0.01) (Figure 6D). IHC further demonstrated upregulated NOTCH3 expression in these tissues (Figure 6E). In contrast, co‐transfection with Lv‐oe‐LINC00958 and miR‐129‐2‐3p agomir led to increased miR‐129‐2‐3p expression, decreased NOTCH3 levels (*p* < 0.05) (Figure 6E), and a reversal of LINC00958‐induced tumor growth (*p* < 0.05) (Figure 6D). Collectively, these findings confirm that LINC00958 promotes EC progression in vivo via the *miR‐129‐2‐3p*/*NOTCH3* axis (Figure 6F).

## 4 Discussion

EC represents one of the most prevalent gynecological malignancies worldwide, with its incidence steadily increasing, particularly in industrialized nations [1]. The combination of elevated disease‐related mortality and a surge in newly diagnosed cases highlights the growing burden of EC on women's health [25]. Despite recent advances in our understanding of EC pathogenesis, many therapeutic approaches remain subject to ongoing debate, reflecting gaps in evidence‐based consensus and clinical efficacy [26]. Therefore, the identification of novel molecular targets and the development of effective therapeutic interventions are urgently needed. This study aimed to investigate the functional contributions of lncRNA LINC00958 and NOTCH3 in driving the malignant progression of EC cells, thereby offering potential targets for therapeutic intervention.

lncRNAs have been recognized as pivotal regulators in tumorigenesis, primarily by modulating the expression of oncogenes through the suppression of specific miRNAs [27]. Among these, LINC00958 has garnered substantial attention for its oncogenic role across diverse malignancies and holds considerable potential as a novel biomarker and therapeutic target [9]. Notably, LINC00958 has been shown to regulate the malignant phenotypes of multiple cancers. In colorectal cancer, its downregulation promotes apoptosis and inhibits malignancy via targeting miR‐3619‐5p [28], while in cervical cancer, it facilitates metastasis and proliferation via the miR‐625‐5p/LRRC8E axis [29]. In the context of EC, LINC00958 has been shown to promote tumor progression through modulation of the miR‐145‐3p/TCF4 axis [10]. Moreover, it is notably upregulated in EC cell lines and specimens, with elevated expression correlating with poor clinical outcomes. Knockdown of LINC00958 has been shown to inhibit the proliferation, migration, and invasion of EC cells, particularly in Ishikawa and KLE cells [30]. Our findings corroborate these observations by demonstrating that LINC00958 augments the malignant biological behaviors of EC cells, and its downregulation reverses these effects. A previous study further revealed that lncRNA XIST regulates EC progression by functioning as a miR‐129‐2‐3p sponge [14]. In the present study, bioinformatics analysis using the IntaRNA 2.0 database predicted the binding sites between LINC00958 and miR‐129‐2‐3p, which were substantiated through dual‐luciferase assays. To our knowledge, this is the first report confirming LINC00958 as a direct regulator of miR‐129‐2‐3p. In addition, miR‐129‐2‐3p has been shown to act as a tumor suppressor in various cancers; its reexpression impedes colon tumor growth in vivo and attenuates esophageal carcinoma cell proliferation in vitro [31, 32]. In line with these findings, we demonstrated that restoration of miR‐129‐2‐3p expression partially abrogates the tumor‐promoting effects of LINC00958 in EC cells, thereby limiting their invasive and proliferative potential.

To elucidate the mechanism by which LINC00958 regulates EC progression, we explored the potential downstream target of miR‐129‐2‐3p. The NOTCH pathway has been extensively studied for its dual role in tumorigenesis, functioning as either an oncogene or tumor suppressor depending on cellular context [33]. Among its receptors, NOTCH3 has been reported to be significantly overexpressed in EC tissues compared to normal endometrial gland cells and is associated with adverse clinical outcomes [18]. Our findings corroborated this evidence, as EC cells exhibited elevated NOTCH3 expression. Knockdown of LINC00958 resulted in decreased NOTCH3 expression, while its overexpression led to further upregulation. Co‐overexpression of LINC00958 and miR‐129‐2‐3p attenuated NOTCH3 expression, suggesting that miR‐129‐2‐3p negatively regulates NOTCH3. Utilizing the StarBase database, we predicted a direct interaction between miR‐129‐2‐3p and the 3′‐UTR of NOTCH3, which was validated experimentally using dual‐luciferase reporter assays. This mechanistic insight aligns with previous studies where NOTCH3 silencing attenuated malignant phenotypes in bladder and ovarian cancers by restricting proliferation and inducing apoptosis [34, 35]. In concordance, our results revealed that NOTCH3 knockdown mitigated the oncogenic effects of LINC00958 in EC cells, highlighting its role as a functional downstream effector. To substantiate these observations, we conducted in vivo *experiments using a* xenograft tumor model. Mice injected with LINC00958 overexpressing EC cells developed larger tumors with increased proliferative activity. Correspondingly, tumors exhibited upregulated expression of LINC00958 and NOTCH3, and downregulation of miR‐129‐2‐3p. Notably, administration of a miR‐129‐2‐3p agomir attenuated tumor growth, confirming its antagonistic role in LINC00958‐driven tumorigenesis. These findings demonstrate that LINC00958 exerts its pro‐tumorigenic effects by sponging miR‐129‐2‐3p to activate NOTCH3, thereby accelerating EC progression in vivo.

Taken together, our study reveals a novel molecular axis, LINC00958/miR‐129‐2‐3p/NOTCH3, that contributes to the malignant transformation of EC cells. Nevertheless, an important limitation is the absence of in vivo rescue experiments involving LINC00958 overexpression combined with NOTCH3 knockdown, which would provide stronger evidence for causality. In future investigations, we aim to conduct such experiments and explore downstream targets of NOTCH3, particularly its involvement in canonical oncogenic pathways, including the Hippo/YAP, NF‐κB, mTOR, and ERK1/2 signaling pathways.

## Ethics Statement

All experimental procedures involving animals were reviewed and approved by the Animal Ethics Committee of The Affiliated Hospital of Yangzhou University, and were performed in full compliance with the institutional ethical guidelines and the approved protocol.

## Conflicts of Interest

The authors declare no conflicts of interest.

## Data Availability

The data that support the findings of this study are available from the corresponding author upon reasonable request.
